# Trajectories of procrastination among Swedish University students over one academic year: a cohort study

**DOI:** 10.1186/s40359-024-02072-2

**Published:** 2024-10-15

**Authors:** Fred Johansson, Alexander Rozental, Klara Edlund, Margreth Grotle, Ann Rudman, Irene Jensen, Eva Skillgate

**Affiliations:** 1 Department of Health Promoting Science, Sophiahemmet University, Stockholm, Sweden; 2 Centre for Psychiatry Research, Department of Clinical Neuroscience, Karolinska Institutet, & Stockholm Health Care Services, Region Stockholm, Stockholm, Sweden; 3 Department of Health, Education and Technology, Luleå University of Technology, Luleå, Sweden; 4 Department of Clinical Neuroscience, Karolinska Institutet, Stockholm, Sweden; 5 Centre for Intelligent Musculoskeletal Health, Department of Rehabilitation and Health Technology, Oslo Metropolitan University, Oslo, Norway; 6 Research and Communication Unit for MSK Health (FORMI), Division of Clinical Neuroscience, Oslo University Hospital, Norway, Oslo Norway; 7 Department of Caring Sciences, Dalarna University, Falun, Sweden; 8 Unit of Intervention and Implementation Research for Worker Health, Institute of Environmental Medicine, Karolinska Institutet, Stockholm, Sweden

**Keywords:** Procrastination, Perfectionism, University students

## Abstract

**Background:**

Procrastination is common among university students and associated with adverse outcomes such as physical and mental health problems. According to the Temporal motivation theory procrastination may vary over time depending on the temporal proximity to goals and deadlines.

**Aims:**

To determine if mean procrastination levels among university students varies over an academic year, and if trajectories of procrastination are moderated by gender identity, perfectionistic strivings, and/or perfectionistic concerns.

**Sample:**

Swedish university students (*n* = 1410).

**Methods:**

The cohort was followed with web-surveys at four time-points over one academic year (Late semester, Mid semester, After semester, and Early semester). Generalized Estimating Equations were used to estimate mean levels of self-rated procrastination at the different time-points.

**Results:**

We found only small fluctuations in mean procrastination levels over the academic year. Participants with high perfectionistic concerns demonstrated higher mean procrastination levels at all time-points, but neither gender identity, perfectionistic concerns nor perfectionistic strivings affected the slope of the mean procrastination trajectories.

**Conclusions:**

In this cohort of Swedish university students, self-rated procrastination levels were stable over the academic year. Perfectionistic concerns, but not gender identity or perfectionistic strivings, was associated with higher levels of procrastination.

**Supplementary Information:**

The online version contains supplementary material available at 10.1186/s40359-024-02072-2.

## Background

Procrastination refers to our innate tendency to “voluntarily delay an intended course of action despite expecting to be worse off for the delay” [1], reflecting the irrational and rash behavior of postponing a task or commitment even though it will result in negative consequences. This is different from strategic delay and delay caused by factors beyond our control as it goes against better judgment [2]. Procrastination can occur in every life domain but is particularly prevalent in academic settings. Research on university students suggests that about half of this population identify themselves as procrastinators and that they procrastinate recurrently [3, 4]. Far from all suffer from procrastination to such a degree that it warrants treatment [5], but a number of investigations nevertheless indicate that a higher level of self-reported procrastination is related to increased symptom severity for various mental health issues. Systematic reviews and meta-analyses with different community samples have demonstrated small correlations with depression [1, 6] and anxiety [6], and several studies have found small correlations with stress [7–10]. Among those who seek help for procrastination, it has also been shown to be negatively associated with quality of life [11]. Although the association with academic achievement is quite weak [1, 12], and a few studies suggest that some forms of procrastination could increase performance and foster creativity [13, 14], it is generally considered to be negative and associated with maladaptive coping strategies [15].

In a recent study by Rozental et al. [16], university students were grouped according to their severity level of procrastination, based on the responses to different self-report measures. The “severe procrastinators” perceived their behavior as more problematic and were more inclined to seek professional help for procrastination than the less severe procrastinators. Furthermore, the severe procrastinators considered themselves to be more negatively affected by procrastination on all life domains, particularly concerning work/studies. They also scored higher on self-report measures of depression, anxiety, and stress, and lower on quality of life, and had a higher proportion of self-reported psychiatric disorders [16]. Further, a recent cohort study found that procrastination among university students was associated, although weakly, with several health outcomes nine months later, including mental health, pain and health behaviors, after controlling for a large set of potential confounders and baseline outcome levels [17].

From a theoretical standpoint procrastination can be conceived as a self-regulatory failure that is often explained by the interaction of four variables; the *value* associated with completing an activity, the *expectancy* to achieve this value, the *time* that remains to attain the desired value, and our level of *impulsivity* (i.e., sensitivity to delay) [18]. According to this model, the Temporal Motivation Theory (TMT), *time* is of particular importance as future goals are valued less, “discounted”, than those that are within our reach, a phenomenon known as hyperbolic discounting [19]. Similarly, *impulsivity* suggests that access to more immediate gratifications will result in preference reversal where we likely will choose proximal over distal rewards once we pursue an activity. Hence, the proximity of *value* is key to initiate a task or commitment, which is presumed to affect procrastinators more than others [1]. Assuming such a hyperbolic trajectory, our motivation should increase, while procrastination decrease, slowly and incrementally as we move closer to our deadline. This is believed to be especially cumbersome in contexts that have set working schedules (i.e., fixed intervals), such as an academic setting where assignments and exams are to be completed at the end of the semester.

Few studies have tested the hyperbolic nature of procrastination in real life using longitudinal study designs. Steel, Brothen, and Wambach [20] investigated 152 university students with regard to procrastination and performance on several occasions during an introductory course in psychology. The results showed that procrastinators and non-procrastinators did not differ in terms of their intentions, but that procrastinators exhibited greater difficulties acting upon them. In essence, procrastinators did less work in the beginning of the course, only to find themselves having to catch up later on, which in turn affected their performance on the final exam negatively. Steel, Svartdal, Thundiyil, and Brothen [21] used a similar approach with 171 university students, measuring procrastination and performance also during an introductory course in psychology. The findings demonstrated that goal-pursuit matched a hyperbolic curve, with procrastinators generally doing fewer assignments per day, except towards the end: “On the final day, maximal procrastinators are showing a very sharp curve, completing over nine assignments on average, which is eleven times the highest average daily output for non-procrastinators.” [21]. Similar evidence has been demonstrated by Yerdelen, McCaffrey, and Klassen [22] among 182 university students drawn from an educational psychology participant pool and followed over one semester. Meanwhile, Moon and Illingworth [23], who recruited 303 university students from an introductory course in psychology, found a different curvilinear trend, where procrastination increased rather than decreased over a period of one semester. However, in this latter study, a proxy for procrastination was used instead of a self-report measure (i.e., hours allocated to studying), and the semester included several deadlines rather than one single end point.

Albeit providing some evidence for a hyperbolic trajectory among procrastinators, previous research is also limited by including relatively few and homogeneous participants (mostly university students in psychology) and employing short time series (ranging from 11 weeks to one semester). Whether similar results are possible to obtain among a larger and more diverse sample and over longer periods is unclear, warranting further investigations using a longitudinal study design. Moreover, it remains to be seen what variables affect the tendency to delay a task or commitment over time. To date, most studies on this issue have been cross-sectional, demonstrating a robust relationship between such personality traits as impulsivity and procrastination [1, 6]. However, a few notable exceptions exist. Steel, Brothen, and Wambach [20] failed to find relationships with facets of personality, mood, and affect at baseline, and trends of procrastination over time. Meanwhile, Steel et al. [21] demonstrated that self-rated distractibility (i.e., being unable to suppress needs and desires) was positively related to procrastination, as was fear of failure (but when also accounting for lack of energy this association was reduced). Lastly, Yerdelen, McCaffrey, and Klassen [22] were unable to demonstrate that baseline academic self-efficacy affected changes in procrastination over the semester.

Following the scarcity of research employing a longitudinal study design in investigating the hyperbolic trajectory of procrastination, further studies should be performed. This also includes exploring other possible variables to understand what might affect trajectories of procrastination. Females typically have a developmental advantage over males in terms of inhibition and self-control when entering the university [1, 6]. This advantage could potentially influence the trajectory of procrastination, as impulsivity is one of the factors hypothesized to influence procrastination trajectories in the TMT [18]. Furthermore, perfectionism is also relevant to examine as it is characterized by being concerned over making mistakes and setting high standards [24], which can lead to procrastination and be particularly problematic in an academic setting. Perfectionism is a multidimensional construct consisting of perfectionistic concerns (i.e., being self-critical and overly concerned about not living up to one’s own or others’ standards), as well as perfectionistic strivings (i.e., striving to be perfect and having high expectations). Differentiating the two has demonstrated that perfectionistic concerns is positively correlated with procrastination, while perfectionistic strivings, on the other hand, is negatively correlated with procrastination [25], suggesting that it is the more anxiety-related aspects of perfectionism that leads to postponement and may be of interest to examine in relation to the tendency to procrastinate over time [16]. Theoretically, high perfectionistic strivings may affect the value assigned to completing an activity (leading to less procrastination when the deadline is far away), while perfectionistic concerns may be more closely related to the expectancy of completing the activity (leading to more procrastination when deadlines are far). This is highly speculative, however, as research of the effect of perfectionism on procrastination trajectories is lacking. A better understanding of the assumed hyperbolic trajectory of procrastination in an academic setting and possible moderating variables could provide insights into when and for whom interventions targeting procrastination may have the greatest effect.

The purpose of the current study was to investigate trajectories of procrastination at four time points over one year to determine whether mean levels of procrastination follow a hyperbolic trajectory, as well as to examine if gender identity and perfectionism moderate the trajectory. As higher education in Sweden typically employs final exams and assignments at the end of each semester, it is hypothesized that mean procrastination levels will be higher early on during the semester and less distinguished towards the end of the semester (i.e., a decreasing trend), in accordance with present theoretical assumptions [18], and previous findings [20–22]. Further, we hypothesize that procrastination will be higher among males and participants with higher perfectionistic concerns, but lower among participants with high perfectionistic strivings. Given lack of prior knowledge, we have no a-priori hypotheses on how gender identity and perfectionism may modify the trajectory of procrastination levels over the academic year.

## Methods

### Design and study population

The current study leveraged data from the Sustainable University Life cohort (SUN) (http://clinicaltrials.gov/ ID: NCT04465435), that include university students at undergraduate or masters’ levels with at least two semesters left of their academic studies. Participants were recruited from eight universities in the greater Stockholm area (the capitol and most populated region in Sweden, with 2.34 million inhabitants) and Örebro, with data collection ongoing between August 2019 to December 2021. The targeted universities constitute a convenience sample aiming to represent a variety of different educational programs

Invited university students received information about the study through in-class presentations by study staff and/or received an email with information and an access link to the web-survey. Information about the study was also distributed through relevant forums and social media (e.g., student unions and information screens on campus). Participants agreeing to participate were followed with web-surveys every three months for one year. The study was approved by the Swedish Ethical Review Authority (2019–03276, 2020–01449, 2022–01435-02) and all participants provided informed consent electronically before entering the study. More information on the data collection and methodology is available in the study protocol [26].

### Data organization

**Fig. 1:**
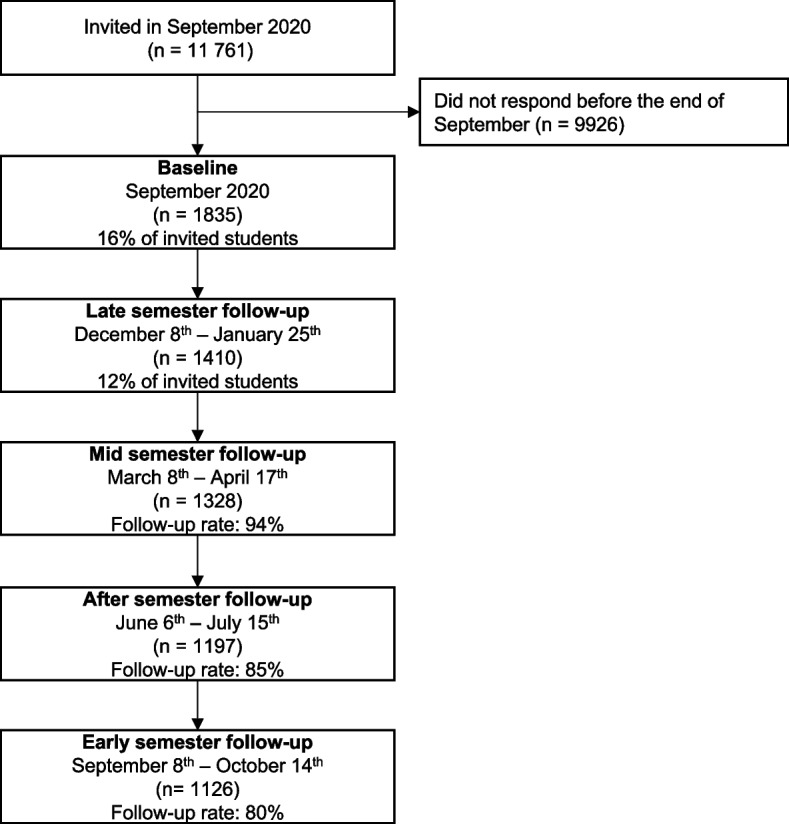
Flowchart of the inclusion of participants. The follow-up rate is calculated based on the 1410 participants in the analytic sample

The current study was restricted to participants entering the SUN-study during September 2020, in order to create a sample that was measured at comparable times during the semester. As procrastination was not measured at the baseline assessment, the sample includes only participants who responded to at least the first follow-up survey, labelled “Late semester” (Fig. 1). This first follow-up occurred between December 8, 2020, and January 25, 2021, and was defined as “Late semester”; the second follow-up period occurred between March 8, 2021, and April 17, 2021, and was defined as “Mid-semester”; the third follow-up period occurred between June 16, 2021, and July 15, 2021, and was defined as “After semester” and the fourth follow-up period occurred between September 8, 2021, and October 14, 2021, and was defined as “Early semester”. Participants responding after the defined time-periods were excluded from this analysis.

### Measures

Demographic information such as age, type of education, and highest level of parental education was collected in the baseline survey.

### Exposures/moderators

Gender identity was collected in the baseline survey using the question “How do you define your gender identity?”, with response categories “Female”, “Male”, “Other”, and “Do not want to identify with either of these identities”. The two latter categories were collapsed into the category “Other gender” for presentation of the sample in Table 1.

**Table 1:** Baseline characteristics for the full sample and stratified by gender identity, perfectionistic concerns, and perfectionistic strivings

	**Study sample**	**Gender identity**^a^		**Perfectionistic concerns**		**Perfectionistic strivings**	
	(*n* = 1410)	Female (*n* = 795)	Male (*n* = 606)	High (*n* = 698)	Low (*n* = 712)	High (*n* = 686)	Low (*n* = 724)
Procrastination, *M* (*SD*)^b^	13.4 (5.3)	13.2 (5.3)	13.5 (5.3)	14.4 (5.4)	12.3 (5.0)	13.2 (5.4)	13.5 (5.2)
Age, *M* (*SD*)	23.2 (5.1)	23.5 (5.5)	22.8 (4.7)	23.1 (4.5)	23.3 (5.7)	22.9 (4.7)	23.5 (5.5)
Gender							
Female, *n* (*%*)	795 (56%)	-	-	440 (63%)	355 (50%)	398 (58%)	397 (55%)
Male, *n* (*%*)	606 (43%)	-	-	255 (37%)	351 (49%)	286 (42%)	320 (44%)
Other gender, *n* (*%*)^c^	9 (1%)	-	-	-	-	-	-
Education type							
Medical/health, *n* (*%*)	240 (17%)	188 (24%)	51 (8%)	117 (17%)	123 (17%)	108 (16%)	132 (18%)
Technical, *n* (*%*)	944 (67%)	438 (55%)	500 (83%)	461 (66%)	483 (68%)	472 (69%)	472 (65%)
Social science/Humanities, *n* (*%*)	224 (16%)	169 (21%)	53 (9%)	119 (17%)	105 (15%)	105 (15%)	119 (16%)
Other, *n* (*%*)^c^	2 (0%)	-	-	-	-	-	-
Highest parental education level							
University, *n* (*%*)	1026 (73%)	573 (72%)	448 (74%)	503 (72%)	523 (73%)	494 (72%)	532 (73%)
Below university, *n* (*%*)	384 (27%)	222 (28%)	158 (26%)	195 (28%)	189 (27%)	192 (28%)	192 (27%)
Place of birth							
Sweden, *n* (*%*)	1102 (78%)	626 (79%)	469 (77%)	544 (78%)	558 (78%)	519 (76%)	583 (81%)
Nordic countries, *n* (*%*)	23 (2%)	17 (2%)	6 (1%)	8 (1%)	15 (2%)	7 (1%)	16 (2%)
Europe, *n* (*%*)	98 (7%)	52 (7%)	46 (8%)	47 (7%)	51 (7%)	61 (9%)	37 (5%)
Outside Europe, *n* (*%*)	187 (13%)	100 (13%)	85 (14%)	99 (14%)	88 (12%)	99 (14%)	88 (12%)

^a^Excluding participants stating another gender identity than Female or Male

^b^Measured at the first follow-up labelled “Late term”

^c^Not presented in the crosstabulation due to low cell counts

Perfectionistic concerns and perfectionistic strivings were measured using the Frost Multidimensional Perfectionism Scale (FMPS) [27], subscales Concerns over Mistakes (FMPS-CM) and Personal standards (FMPS-PS). The FMPS-CM consists of nine items and the FMPS-PS of seven items. These are originally rated on a 5-point Likert-scale from 1 (Strongly Agree) to 5 (Strongly Disagree), but in the current study items were rated on a 6-point scale from 0 (Strongly Agree) to 5 (Strongly Disagree). The items are summed to give a total score for each subscale. Cronbach’s α at baseline was 0.90 for the FMPS-CM and 0.87 for the FMPS-PS in this sample. We applied a median split of both scales to create “high” and “low” groups on the perfectionism variables.

### Outcome

Procrastination was measured using five items from the Swedish translation of the Pure procrastination scale (PPS) [11]. The items were rated on a Likert-scale ranging from 1 (“Very rarely or does not represent me”) to 5 (“Very often or always represents me”) and summed to give a total procrastination score ranging 5–25. This short version of the PPS includes items 4–8 from the full PPS. Items 4–8 were chosen as our measure of procrastination since they have shown adequate psychometric properties when used in non-clinical samples, including scalar invariance between males and females [28], which was deemed important in relation to our research questions. These five items reflect the dimension “implemental or irrational delay” and excludes items reflecting “decisional procrastination” (items 1–3) and “timeliness/promptness” (items 9–12). Therefore, this short scale reflects the acratic nature of procrastination, while excluding aspects that are more related to decision-making and being less affected by cultural differences concerning punctuality [28]. Cronbach’s α was 0.91 at the “Late term” follow-up in this sample.

### Statistical analyses

Baseline characteristics of the sample are presented in as means and standard deviations or as counts  and percentages, both for the full sample and stratified by the moderating variables in Table 1.

The trajectory of mean procrastination levels over the academic year was estimated using generalized estimating equations (GEE) with unstructured working correlation matrices, identity link functions and gaussian error distributions, with time-of-semester treated as a categorical variable. Differences in trajectories between gender identity and level of perfectionism were assessed by building models that included interaction terms between these variables and time-of-semester, with a separate model for each of the moderating variables.

No covariates were used in the analyses of procrastination levels over time for the full group, since the effect of time-of-semester is unlikely to be affected by any other factors and thereby not subject to confounding. Nor were any other covariates included in the moderation analyses of gender identity, as any covariates were likely to be on the causal pathway. Participants stating another gender identity than female or male were excluded from this analysis (n = 9). Models examining the interaction between the two respective perfectionism dimensions and time of semester, were adjusted for age, gender identity (female, male, or other), highest level of parental education (university level or below university level), and place of birth (Sweden, Nordic countries, Europe, or Outside Europe), as these were assumed to potentially confound the moderation effects of the perfectionism dimensions.

**Fig. 2:**
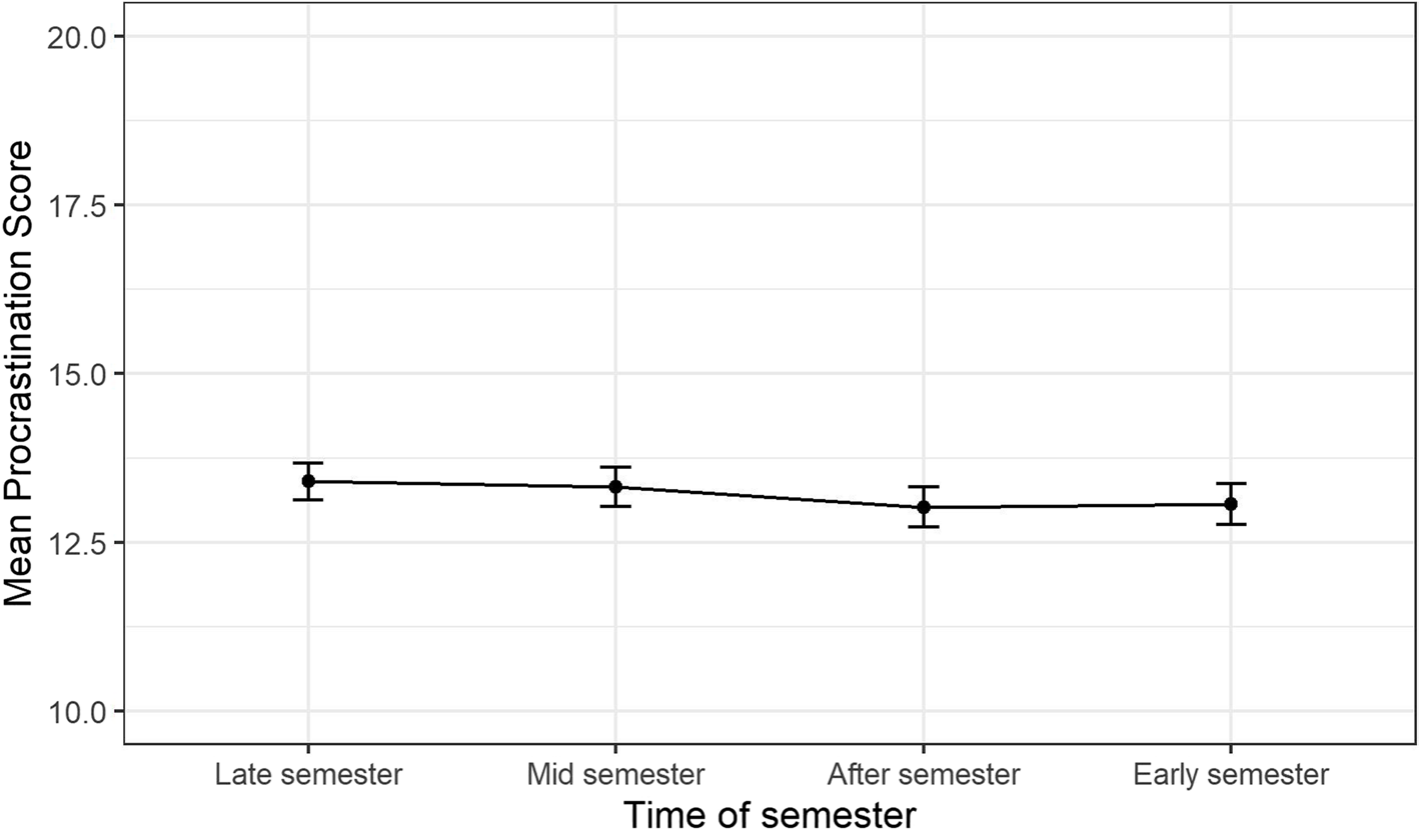
Estimated means of procrastination over different parts of the academic year with 95% confidence intervals

**Fig. 3:**
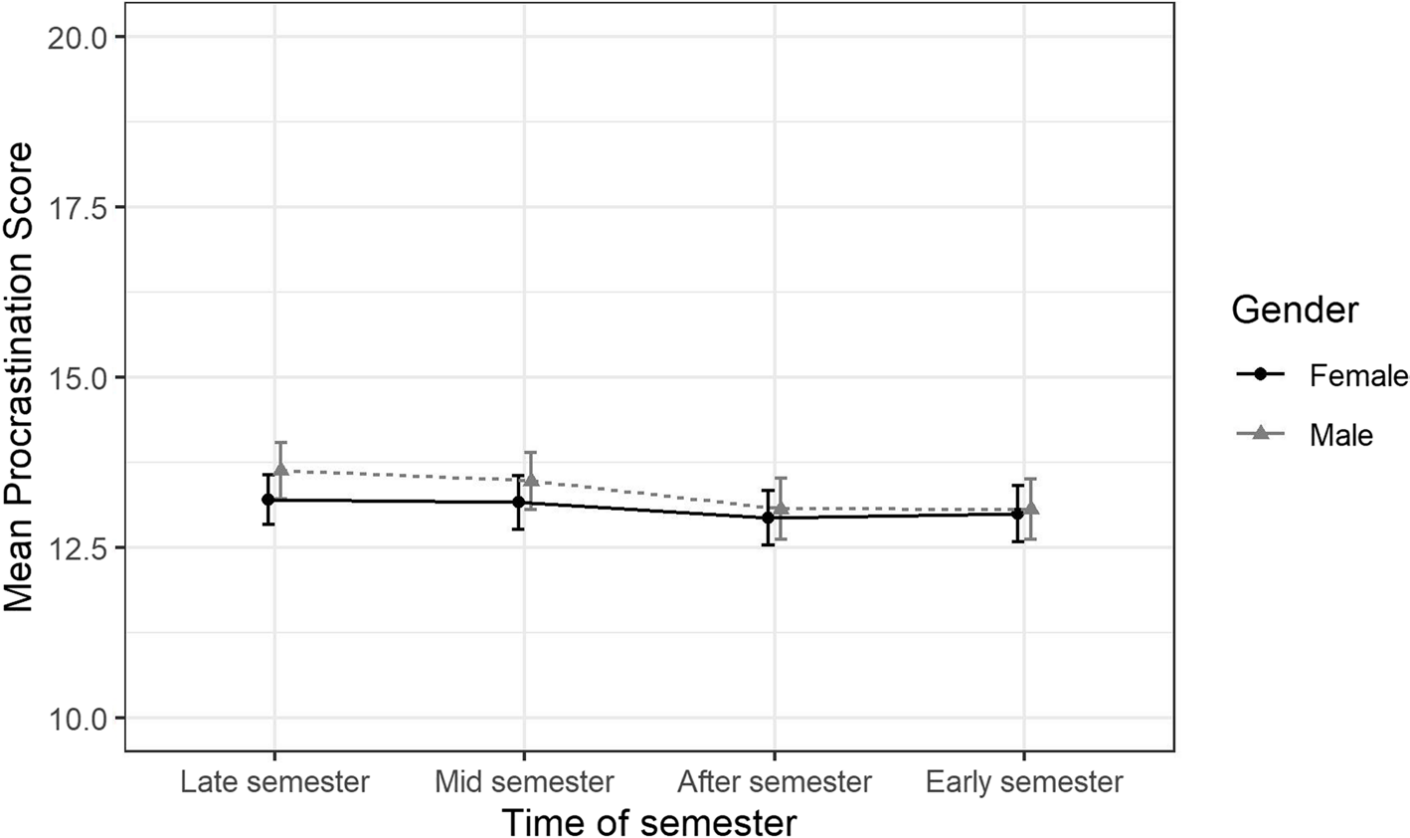
Estimated means of procrastination stratified by gender identity, over different parts of the academic year along with 95% confidence intervals

**Fig. 4:**
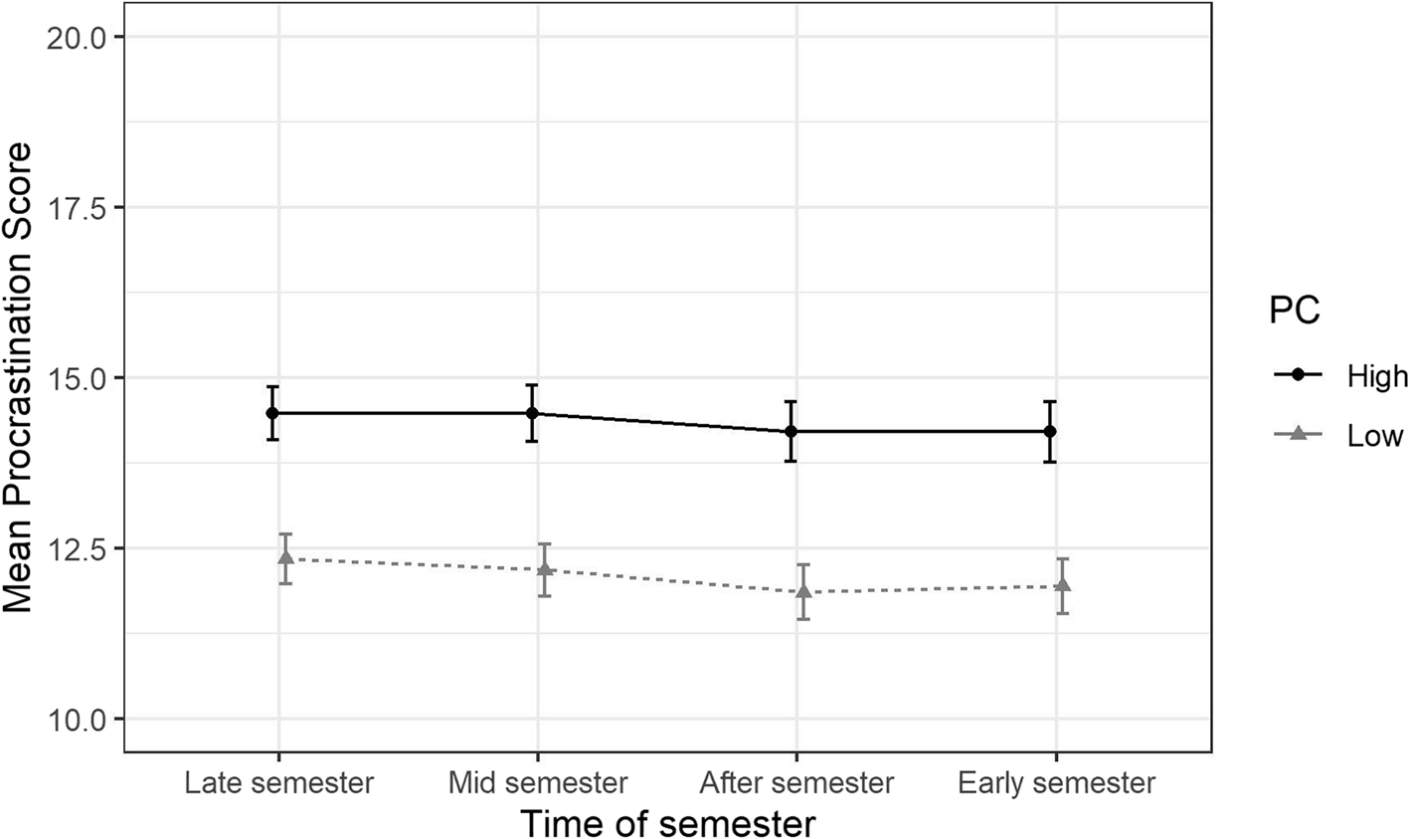
Estimated means of procrastination stratified by perfectionistic concerns (PC), over different parts of the academic year along with 95% confidence intervals. The estimated means are adjusted for and averaged over age, gender identity, highest level of parental education, and place of birth

**Fig. 5:**
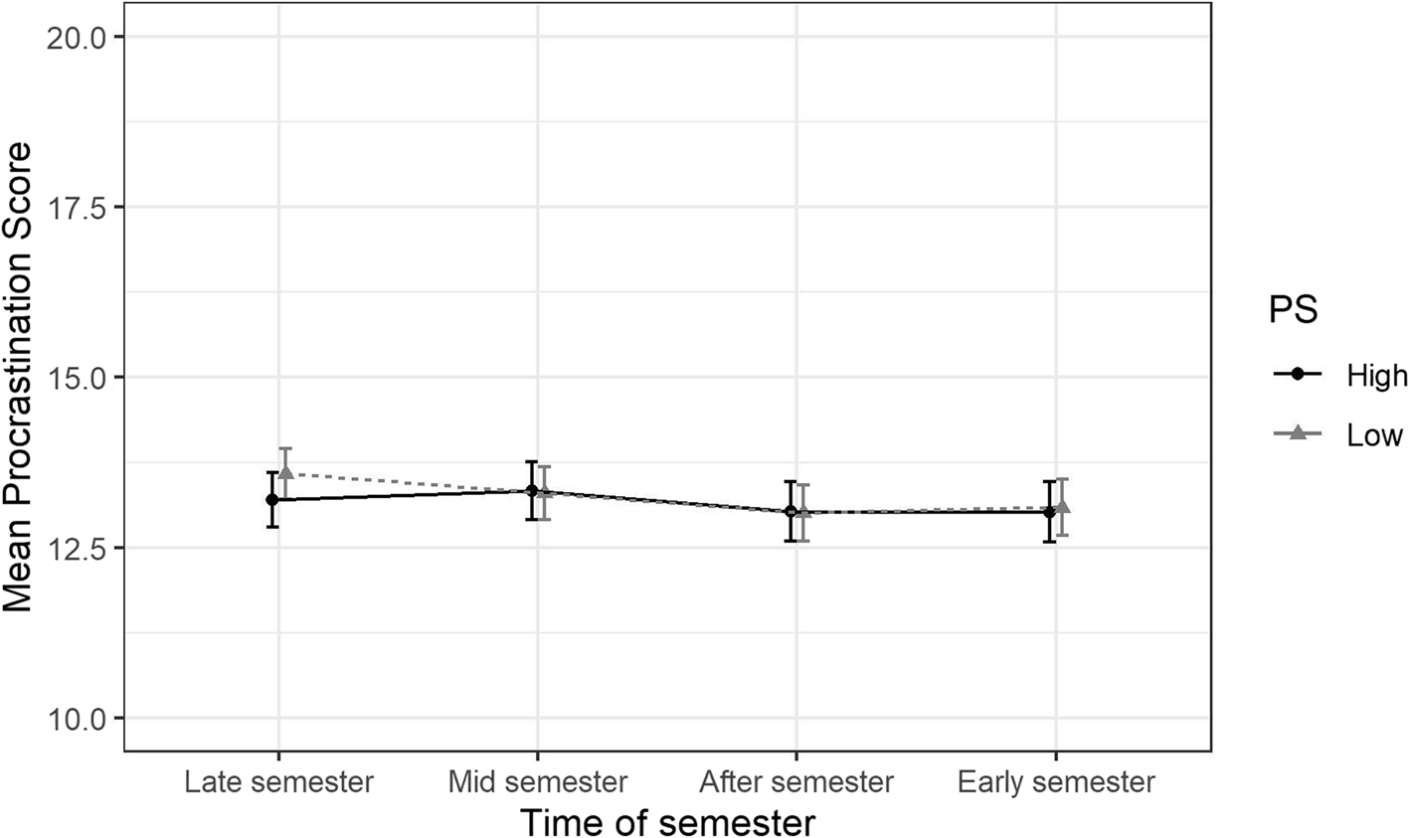
Estimated means of procrastination stratified by perfectionistic strivings (PS), over different parts of the academic year along with 95% confidence intervals. The estimated means are adjusted for and averaged over age, gender identity, highest level of parental education, and place of birth

Results from these analyses are presented as estimated means and presented along with 95% Confidence Intervals (CI) in Figs. 2, 3, 4, 5 and Supplemental eTable 1. The overall differences in trajectories between the exposure levels (gender identity, perfectionistic strivings, and perfectionistic concerns) were tested by using ANOVA-tests on the interaction terms between the exposure variables and the time-of-semester variable at an α-level of 0.05.

To assess the effect of loss to follow-up we compared the analyses of the full sample to complete case analyses. This sensitivity analysis is performed to assess the potential bias in the mean levels of procrastination over time that may arise if participants with higher levels of procrastination are lost to follow-up more frequently compared to participants with lower levels of procrastination. These results are presented in eTable2.

All analyses were performed using R version 4.1.2.

## Results

A total of 11 761 university students were invited to participate in the SUN-study during September 2020, and 1835 joined the study before the end of this month. Of those entering, 1410 responded to the “Late semester” follow-up in December/January, where procrastination was first measured and were included in our sample. Of the 1410 participants included in the sample, 94% (*n* = 1328) responded to the “Mid-semester” follow-up, 85% (*n* = 1197) responded to the “After semester” follow-up, and lastly, 80% (*n* = 1126) responded to the “Early semester” follow-up (Fig. 1).

The mean age of the sample was 23 years (*SD* = 5), 56% were females, the majority was studying at technical education programs (67%) and most had at least one parent with university education (73%) (Table 1). Mean (SD) observed procrastination levels at each time-point were: 13.4 (5.3) at Late-Semester, 13.3 (5.6) at Mid-Semester, 12.9 (5.7) After-Semester and 13.0 (5.6) at Early-semester.

The trajectory of mean procrastination score in the full sample is presented in Fig. 2. The estimated mean procrastination score at the “Late semester” follow-up was 13.4 (95% CI [13.1, 13.7]). The slope of the mean procrastination score was -0.1 (95% CI [-0.3, 0.1]) points at the “Mid semester” follow-up, -0.4 (95% CI [-0.6, -0.2]) points at the “After semester” follow-up, and -0.3 (95% CI [-0.6, -0.1]) points at the “Early semester” follow-up. Estimated mean levels at each time-point are presented in Fig. 2 and eTable1.

The estimated trajectories of mean procrastination scores for males and females are presented in Fig. 3 and eTable 1. At the Late term follow-up, the difference in mean procrastination score between males and females was 0.4 (95% CI [-0.1, 1.0]). The difference in the slopes of procrastination scores over time between males and females were -0.1 (95% CI [-0.5, 0.3]) at the “Mid semester” follow-up, -0.3 (95% CI [-0.7, 0.2]) at the “After semester” follow-up, and -0.4 (95% CI [-0.8, 0.1]) at the “Early semester” follow-up. An ANOVA of the interaction terms between gender identity and time-of-semester showed that there were no significant differences in the slopes of the trajectories between males and females (*p* = 0.42).

Adjusted trajectories of mean procrastination scores for the groups with high and low levels of perfectionistic concerns are presented in Fig. 4 and eTable 1. The adjusted difference between the groups was -2.1 (95% CI [-2.7, -1.6]) at the “Late semester” follow-up. The difference in slopes between the groups were -0.2 (95% CI [-0.6, 0.2]) at the “Mid semester” follow-up, -0.2 (95% CI [-0.7, 0.2]) at the “After semester” follow-up, and -0.1 (95% CI [-0.6, 0.3]) at the “Early semester” follow-up. An ANOVA of the interaction terms between perfectionistic concerns and time-of-semester showed no significant overall difference in slopes over time between the groups (*p* = 0.76), after accounting for the other covariates.

Adjusted trajectories of mean procrastination scores for the groups with high and low perfectionistic strivings are shown in Fig. 5 and Supplemental eTable 1. At the “Late semester” follow-up, the adjusted difference in mean procrastination score between the low and the high group was 0.4 (95% CI [-0.2, 0.9]). The difference in slopes between the groups were -0.4 (95% CI [-0.8, 0.0]) at both the “Mid semester” follow-up and at the “After semester” follow-up, and -0.3 (95% CI [-0.8, 0.1]) points at the “Early semester” follow-up. An ANOVA of the interaction terms demonstrated that there was no significant interaction between perfectionistic strivings and time-of-semester overall (*p* = 0.16), after accounting for the other covariates.

Sensitivity analyses were performed by restricting the sample to the 981 participants who responded at all follow-ups (Supplemental eTable 2). The results were very similar to the main analyses, with the estimated means differing by a maximum of 0.3 points between the main analyses and the sensitivity analyses.

## Discussion

The current study assessed mean levels of procrastination among university students in Sweden at four time-points over the semesters to determine the trajectory of mean procrastination over the academic year. Overall, mean levels of self-rated procrastination were stable over time, demonstrating very little fluctuation over the academic year. Mean procrastination levels were slightly higher late in the semester compared to early in semester and after the semester, thus contradicting our hypothesis of a hyperbolic trajectory, but these differences were only 0.4 points or less on the procrastination scale ranging 5–25. This finding differs from prior research, which generally indicates a decreasing trend of procrastination towards the end of a semester [20, 21], albeit with one exception that instead found an increase [22]. This discrepancy in relation to prior research might be due to several reasons. First, the current study only used items 4–8 (implemental or irrational delay) on the PPS, thereby omitting the other two factors, i.e., decisional procrastination (items 1–3) and lateness/timeliness (items 9–12). Investigating only implemental or irrational delay was deemed appropriate as it represents the more acratic nature of procrastination than the other two factors [28]. However, using all items of the PPS might have revealed a different trend and may be used in future research to assess the trajectory of procrastination for both the full measure and each of the subscales. Second, implemental delay should primarily be affected by contextual antecedents, such as when trying to execute goal-directed behavior (e.g., studying for an upcoming exam). This was difficult to capture in the current study as assessment were made at several predetermined time-points rather than at specific curricular deadlines (e.g., handing in a course assignment). Moving forward, research on the potential hyperbolic nature of procrastination ought therefore to use a similar study design as Steel et al. [18], who explored procrastination in close relation to specific tasks during a course rather than a complete semester. Steel et al. [21] also argue that this type of research should preferably be made using much more frequent measurement points, such as implementing Ecological Momentary Assessment.

Self-reported procrastination, as used in the current study, may also differ somewhat from actual behavior, particularly when being measured at certain time-points. It is thus unclear whether the scores over the semesters reflect overt behavior. Previous research suggest that self-reports are in fact valid assessments of procrastination (c.f., [29]). However, studies like these usually correlate such measures with objective outcomes. To what extent the results of the current study correspond to real-life procrastination by the university students, as in experiencing difficulties initiating study-relevant activities or meeting deadlines, is unclear but could have been examined using grade point averages or course completion rates. Steel, Brothen, and Wambach [20], for example, found a negative relationship between self-reported procrastination and performance on the final exam. Likewise, Steel et al. [21] were able to demonstrate that the number of completed assignments increased dramatically closer to the end of the course. Future studies should therefore use a combination of assessments, both self-report measures and objective outcomes, to explore procrastination among university students.

As for gender identity, male university students were not more prone to procrastinate in the current study, and showed a similar trajectory as female students, contradicting our hypothesis that males would have higher levels of procrastination. However, the influence of gender might not be as pronounced as sometimes argued in the literature [30]. Although a significant difference has been found in systematic reviews and meta-analyses [1, 6], correlations have generally been small (*r*s = -0.05 and -0.08) and may not be of relevance in real-world situations. Yet, it remains to be seen if gender identity plays a role in moderating the level of procrastination in certain age groups, such as among high-school or college students, where a developmental advantage for females could be more evident.

One finding of the current study that helps shed some new light on our understanding of procrastination is the relationship with perfectionism. From a clinical point of view, perfectionism is often mentioned as a predisposing factor to procrastination that should be targeted in psychological treatment [5]. Still, research has long been unable to support such a relationship [1], which might have been due to not differentiating between perfectionistic concerns and perfectionistic strivings [25]. Our results showed that university students in the current study who exhibited higher levels of self-reported perfectionistic concerns did procrastinate to a greater degree across all of time-points of the academic year, in line with our hypothesis and some prior findings [16, 25], although with no clear differences in trajectories between the groups. Our results imply that perfectionistic concerns are related to elevated procrastination levels across the academic year, regardless of the time of the semester. It is therefore possible that perfectionistic concerns are more closely related to a general tendency to procrastinate, rather than levels of procrastination at specific time-points in relation to a deadline. The failure to find differences in trajectories may however also reflect the fact that we were not able to measure academic deadlines directly, as noted above. Meanwhile, perfectionistic strivings did not seem to play a role in their tendency to procrastinate, which was in contrast to our hypothesis and to the negative correlation reported by Sirois [25]. Further research needs to be performed regarding both perfectionism dimensions and their relationship with procrastination. It is nevertheless reasonable to expect an influence of perfectionistic concerns on performance and academic achievements as it characterized by being highly self-critical of oneself and anxious about one’s performance. This may be particularly relevant among those university students who lack self-efficacy and the skills necessary for setting up successful study routines.

It is important to note that the findings in the current study do not preclude assessments of procrastination to be used as a way of identifying those university students that may warrant some support. High scores on the PPS still has a negative relationship to different aspects of well-being [16, 17], suggesting that monitoring procrastination by self-reports is a feasible way to detect those who struggle in an academic setting and provide effective interventions, such as cognitive behavior therapy administered via the Internet or in a group setting [31]. Similarly, this can be combined with a measure of perfectionism to detect those university students who are also overly concerned about their performance and might need some additional help. Lastly, given the fact that a lot of university students do procrastinate and perceive their behavior as problematic [16], teachers should consider how they can set up a study environment that prevents, or at least minimizes, procrastination from occurring. In a review by Svartdal et al. [32], several relatively straightforward examples are provided. This includes such aspects as creating more tangible subgoals instead of relying on distal deadlines during a course, building self-efficacy by regular feedback, using stimulus control (e.g., removing distracting elements), and teaching basic study skills.

### Strengths and limitations

One of the strengths of the current study is that we were able to include a large and diverse sample of Swedish university students and follow them with high response-rates over time. High response-rates are important, since drop-out, especially if this was overrepresented among those with high procrastination levels, could bias the estimation of mean procrastination levels. The diversity of the sample, together with the naturalistic setting in which data was collected, increase our confidence that the results apply to real world situations. We also conducted sensitivity analyses using only complete cases, that indicated that loss to follow-up did not affect our estimated trajectories. A limitation of the current study is that our sample, although diverse, is not representative of the overall student population. First, only 12% of the invited students were included in this sample, which was partly due to restricting the sample to participants who entered before the end of September 2020. Further, our sample contains a majority of technical students, which is not representative of Swedish university students overall. For instance, these students may differ in terms of scheduled education and timing of exams, which could affect trajectories of procrastination. Thus, even with a diverse sample and a naturalistic data collection, we cannot be certain that our results generalize to the overall student population. Further, gender identity is often aligned with sex assigned at birth and influenced by gender norms, on which we have no data. This precludes conclusions on the relative importance of gender identity vs sex or gender in relation to procrastination. Finally, we are limited by our design where procrastination was measured at pre-determined time-points, rather than in relation to actual academic deadlines. This design limits our ability to comprehensively evaluate the TMT.

## Conclusions

We found that mean levels of procrastination, specifically the factor implemental delay, were stable over the academic year and did not follow the hypothesized hyperbolic trajectory in this cohort of Swedish university students. The shape of the trajectory was not influenced by gender identity, perfectionistic concerns, or perfectionistic strivings. Students with high perfectionistic concerns did, however, show higher levels of procrastination throughout the academic year, while procrastination levels were similar with regards to gender identity and perfectionistic strivings.

## Supplementary Information

Supplementary Material 1.

## Data Availability

The dataset analysed in the current study is not publicly available due to secondary confidentiality and privacy of the participants.

## Abbreviations

ANOVA: Analysis of variance
CI: Confidence interval
FMPS-CM: Frost multi-dimensional perfectionism scale, concerns over mistakes subscale
FMPS-PS: Frost multi-dimensional perfectionism scale, personal standards subscale
GEE: Generalized estimating equations
PPS: Pure Procrastination Scale
SUN: Sustainable University Life study

## References

[1] Steel P. The nature of procrastination: a meta-analytic and theoretical review of quintessential self-regulatory failure. Psychol Bull. 2007;133(1):65–94. 17201571 10.1037/0033-2909.133.1.65

[2] Klingsieck KB. Procrastination: when good things don’t come to those who wait. Eur Psychol. 2013;18:24–34.

[3] Day V, Mensink D, O’Sullivan M. Patterns of academic procrastination. J Coll Read Learn. 2000;30(2):120–34.

[4] Grunschel C, Schopenhauer L. Why are students (Not) motivated to change academic procrastination?: an investigation based on the transtheoretical model of change. J Coll Stud Dev. 2015;56(2):187–200.

[5] Rozental A, Carlbring P. Understanding and treating procrastination: a review of a common self-regulatory failure. Psychology. 2014;5(13):1488–502.

[6] van Eerde W. A meta-analytically derived nomological network of procrastination. Personal Individ Differ. 2003;35:1410–8.

[7] Stead R, Shanahan MJ, Neufeld RWJ. “I’ll go to therapy, eventually”: procrastination, stress and mental health. Personal Individ Differ. 2010;49(3):175–80.

[8] Sirois FM, Melia-Gordon M, Pychyl T. ‘I’ll look after my health, later’: an investigation of procrastination and health. Personal Individ Differ. 2003;35:1167–84.

[9] Sirois FM. “I’ll look after my health, later”: a replication and extension of the procrastination–health model with community-dwelling adults. Personal Individ Differ. 2007;43(1):15–26.

[10] Beutel ME, Klein EM, Aufenanger S, Brähler E, Dreier M, Müller KW, et al. Procrastination, distress and life satisfaction across the age range - a German representative community study. PLoS One. 2016;11(2):e0148054. 26871572 10.1371/journal.pone.0148054 PMC4752450

[11] Rozental A, Forsell E, Svensson A, Forsström D, Andersson G, Carlbring P. Psychometric evaluation of the Swedish version of the pure procrastination scale, the irrational procrastination scale, and the susceptibility to temptation scale in a clinical population. BMC Psychol. 2014;2(1):54. 25566392 10.1186/s40359-014-0054-z PMC4269972

[12] Kim KR, Seo EH. The relationship between procrastination and academic performance: a meta-analysis. Personal Individ Differ. 2015;82:26–33.

[13] Chun Chu AH, Choi JN. Rethinking procrastination: positive effects of ‘active’ procrastination behavior on attitudes and performance. J Soc Psychol. 2005;145(3):245–64. 15959999 10.3200/SOCP.145.3.245-264

[14] Shin J, Grant AM. When putting work off pays off: the curvilinear relationship between procrastination and creativity. Acad Manage J. 2021;64(3):772–98.

[15] Sirois FM, Kitner R. Less adaptive or more maladaptive? A meta–analytic investigation of procrastination and coping. Eur J Personal. 2015;29(4):433–44.

[16] Rozental A, Forsström D, Hussoon A, Klingsieck KB. Procrastination among university students: differentiating severe cases in need of support from less severe cases. Front Psychol. 2022;13:783570. 10.3389/fpsyg.2022.783570 PMC8965624 35369255

[17] Johansson F, Rozental A, Edlund K, Côté P, Sundberg T, Onell C, et al. Associations between procrastination and subsequent health outcomes among university students in Sweden. JAMA Netw Open. 2023;6(1):e2249346–e2249346. 36598789 10.1001/jamanetworkopen.2022.49346 PMC9857662

[18] Steel P, König CJ. Integrating theories of motivation. Acad Manage Rev. 2006;31(4):889–913.

[19] Ainslie G, Haslam N. Hyperbolic discounting. In: Choice over time. New York, NY, US: Russell Sage Foundation; 1992. p. 57–92.

[20] Steel P, Brothen T, Wambach C. Procrastination and personality, performance, and mood. Personal Individ Differ. 2001;30(1):95–106.

[21] Steel P, Svartdal F, Thundiyil T, Brothen T. Examining procrastination across multiple goal stages: a longitudinal study of temporal motivation theory. Front Psychol. 2018;9:327. 29666590 10.3389/fpsyg.2018.00327 PMC5891720

[22] Yerdelen S, McCaffrey A, Klassen R. Longitudinal examination of procrastination and anxiety, and their relation to self-efficacy for self- regulated learning: latent growth curve modeling. Educ Sci Theory Pract. 2016;16:5–22.

[23] Moon SM, Illingworth AJ. Exploring the dynamic nature of procrastination: a latent growth curve analysis of academic procrastination. Personal Individ Differ. 2005;38(2):297–309.

[24] Shafran R, Cooper Z, Fairburn CG. Clinical perfectionism: a cognitive-behavioural analysis. Behav Res Ther. 2002;40(7):773–91. 12074372 10.1016/s0005-7967(01)00059-6

[25] Sirois FM, Molnar DS, Hirsch JK. A meta-analytic and conceptual update on the associations between procrastination and multidimensional perfectionism. Eur J Personal. 2017;31(2):137–59.

[26] Edlund K, Sundberg T, Johansson F, Onell C, Rudman A, Holm LW, et al. Sustainable UNiversity Life (SUN) study: protocol for a prospective cohort study of modifiable risk and prognostic factors for mental health problems and musculoskeletal pain among university students. BMJ Open. 2022;12(4): e056489. 35379630 10.1136/bmjopen-2021-056489 PMC8980731

[27] Frost RO, Marten P, Lahart C, Rosenblate R. The dimensions of perfectionism. Cogn Ther Res. 1990;14(5):449–68.

[28] Svartdal F, Steel P. Irrational Delay Revisited: Examining Five Procrastination Scales in a Global Sample. Front Psychol. 2017;8:1927. 10.3389/fpsyg.2017.01927 PMC5676095 29163302

[29] Zuber S, Cauvin S, Haas M, Daviet AS, Da Silva CC, Kliegel M. Do self-reports of procrastination predict actual behavior? Int J Methods Psychiatr Res. 2020;29(4):e1843. 32530112 10.1002/mpr.1843 PMC7723175

[30] Steel P, Ferrari J. Sex, education and procrastination: an epidemiological study of procrastinators’ characteristics from a global sample. Eur J Personal. 2013;27(1):51–8.

[31] Rozental A, Bennett S, Forsström D, Ebert DD, Shafran R, Andersson G, et al. Targeting procrastination using psychological treatments: a systematic review and meta-analysis. Front Psychol. 2018;9:1588. 30214421 10.3389/fpsyg.2018.01588 PMC6125391

[32] Svartdal F, Dahl TI, Gamst-Klaussen T, Koppenborg M, Klingsieck KB. How study environments foster academic procrastination: overview and recommendations. Front Psychol. 2020;11:540910. 33224046 10.3389/fpsyg.2020.540910 PMC7667251

