# Supplementary material for: Trajectories of procrastination among Swedish University students over one academic year: a cohort study
Source: BMC Psychol. 2024 Oct 15;12:559. doi: 10.1186/s40359-024-02072-2 (PMC11481787; doi:10.1186/s40359-024-02072-2)
Supplement: Supplementary file 1 — Supplementary Material 1. [file 40359_2024_2072_MOESM1_ESM.pdf]

Supplementary material

Johansson, F., Rozental, A., Edlund, K., Grotle, M., Rudman, A., Jensen, I, and Skillgate, E. **Trajectories of Procrastination among Swedish University Students Over One Academic Year: A Cohort Study**

eTable 1. Estimated mean levels and 95% confidence intervals of procrastination over time-of-semester for the full sample and stratified by gender, perfectionistic concerns and personal standards

	**Full sample**	**Gender identity**		**Perfectionistic concerns ***		**Personal standards ***	
		Females	Males	High group	Low group	High group	Low group
	(*n* = 1410)	(*n* = 795)	(*n* = 606)	(*n* = 698)	(*n* = 712)	(*n* = 686)	(*n* = 724)
	M (95 % CI)	*M* (95 % CI)	*M* (95 % CI)	*M* (95 % CI)	*M* (95 % CI)	*M* (95 % CI)	*M* (95 % CI)
**Time-of-term**							
Late semester	13.4	13.2	13.6	14.5	12.3	13.2	13.6
	(13.1, 13.7)	(12.8, 13.6)	(13.2, 14.0)	(14.1, 14.9)	(12.0, 12.7)	(12.8, 13.6)	(13.2, 13.9)
Mid semester	13.3	13.2	13.5	14.5	12.2	13.3	13.3
	(13.0, 13.6)	(12.8, 13.6)	(13.1, 13.9)	(14.1, 14.9)	(11.8, 12.6)	(12.9, 13.8)	(12.9, 13.7)
After semester	13.0	12.9	13.1	14.2	11.8	13.0	13.0
	(12.7, 13.3)	(12.5, 13.3)	(12.6, 13.5)	(13.8, 14.6)	(11.4, 12.2)	(12.6, 13.5)	(12.6, 13.4)
Early semester	13.1	13.0	13.1	14.2	11.9	13.0	13.1
	(12.8, 13.4)	(12.6, 13.4)	(12.6, 13.5)	(13.8, 14.6)	(11.5, 12.3)	(12.6, 13.5)	(12.7, 13.5)

*Adjusted for and averaged over age, gender identity, highest level of parental education, and place of birth

eTable 2. Sensitivity analysis including only complete cases. Estimated mean levels and 95 % confidence intervals of procrastination over time-of-term for the full sample of complete cases and stratified by gender, perfectionistic concerns and personal standards.

	**Full sample**	**Gender identity**		**Perfectionistic concerns ***		**Personal standards ***	
		Females	Males	High group	Low group	High group	Low group
	(n=981)	(n = 555)	(n= 419)	(n = 480)	(n = 501)	(n = 466)	(n = 515)
	M (95 % CI)	M (95 % CI)	M (95 % CI)	M (95 % CI)	M (95 % CI)	M (95 % CI)	M (95 % CI)
**Time-of term**							
Late term	13.2	13.0	13.5	14.5	12.1	13.0	13.5
	(12.9, 13.6)	(12.6, 13.5)	(13.0, 14.0)	(14.0, 15.0)	(11.6, 12.5)	(12.5, 13.4)	(13.1, 14.0)
Mid-term	13.2	13.0	13.3	14.5	11.9	13.2	13.2
	(12.8, 13.5)	(12.5, 13.5)	(12.8, 13.9)	(14.0, 15.0)	(11.5, 12.4)	(12.7, 13.6)	(12.7, 13.6)
After term	12.8	12.7	12.9	14.1	11.6	12.8	12.8
	(12.5, 13.2)	(12.2, 13.2)	(12.4, 13.5)	(13.6, 14.6)	(11.2, 12.1)	(12.3, 13.3)	(12.4, 13.3)
Early term	12.8	12.7	12.8	14.0	11.6	12.8	12.8
	(12.4, 13.1)	(12.3, 13.2)	(12.3, 13.3)	(13.5, 14.6)	(11.1, 12.0)	(12.2, 13.3)	(12.4, 13.3)

*Adjusted for and averaged over age, gender identity, highest level of parental education and place of birth
